# The contribution of preclinical magnetic resonance imaging and spectroscopy to Huntington’s disease

**DOI:** 10.3389/fnagi.2024.1306312

**Published:** 2024-02-13

**Authors:** Jean-Baptiste Pérot, Emmanuel Brouillet, Julien Flament

**Affiliations:** ^1^Laboratoire des Maladies Neurodégénératives, Molecular Imaging Research Center, Commissariat à l’Energie Atomique et aux Energies Alternatives, Centre National de la Recherche Scientifique, Université Paris-Saclay, Fontenay-aux-Roses, France; ^2^Institut du Cerveau – Paris Brain Institute – ICM, Sorbonne Université, Paris, France

**Keywords:** Huntington’s disease, ^1^H-MRS, MRI, mouse model, preclinical imaging

## Abstract

Huntington’s disease is an inherited disorder characterized by psychiatric, cognitive, and motor symptoms due to degeneration of medium spiny neurons in the striatum. A prodromal phase precedes the onset, lasting decades. Current biomarkers include clinical score and striatal atrophy using Magnetic Resonance Imaging (MRI). These markers lack sensitivity for subtle cellular changes during the prodromal phase. MRI and MR spectroscopy offer different contrasts for assessing metabolic, microstructural, functional, or vascular alterations in the disease. They have been used in patients and mouse models. Mouse models can be of great interest to study a specific mechanism of the degenerative process, allow better understanding of the pathogenesis from the prodromal to the symptomatic phase, and to evaluate therapeutic efficacy. Mouse models can be divided into three different constructions: transgenic mice expressing exon-1 of human huntingtin (HTT), mice with an artificial chromosome expressing full-length human HTT, and knock-in mouse models with CAG expansion inserted in the murine htt gene. Several studies have used MRI/S to characterized these models. However, the multiplicity of modalities and mouse models available complicates the understanding of this rich corpus. The present review aims at giving an overview of results obtained using MRI/S for each mouse model of HD, to provide a useful resource for the conception of neuroimaging studies using mouse models of HD. Finally, despite difficulties in translating preclinical protocols to clinical applications, many biomarkers identified in preclinical models have already been evaluated in patients. This review also aims to cover this aspect to demonstrate the importance of MRI/S for studying HD.

## 1 Introduction

Huntington’s disease (HD) is the most frequent polyglutamine (polyQ) which is characterized by progressive motor dysfunction, cognitive decline, and psychiatric disturbances. As the quest for effective therapeutic interventions intensifies, the need to unravel the intricate physiopathological mechanisms underlying HD becomes urgently needed. Preclinical models serve as invaluable tools in this pursuit, offering insights into disease progression and facilitating the development of novel treatment strategies. Although not perfectly replicating the human disease, animal models of HD are valuable tools for cutting-edge methodological developments and to explore the potential of new biomarkers related to the HD mutation *in vivo* (Ramaswamy et al., 2007; Ferrante, 2009; Farshim and Bates, 2018).

Among the myriad techniques employed in preclinical research, magnetic resonance imaging (MRI) and Magnetic Resonance Spectroscopy (MRS) have emerged as powerful and versatile tools, providing non-invasive, high-resolution imaging of the brain but also providing relevant clues about potential biological alterations that occurred in the disease time course. Longitudinal examination of HD models over time with MRI/S, along with behavioral studies are likely to represent a real progress to better understand HD physiopathology.

This review aims to explore the field of MRI and MRS applications in mouse models of HD, shedding light on the pivotal role it plays in elucidating the structural, functional, and metabolic alterations associated with the disease. From anatomical imaging to advanced functional and molecular imaging techniques, MRI/S offers a multidimensional approach to unraveling the complexities of HD pathogenesis. By synthesizing findings from a diverse array of studies, we aim to provide a comprehensive overview of how MRI/S has been employed to characterize the wide variety of mouse models of HD. In order to be as exhaustive as possible, we listed all papers found in a PubMed research using the search term “Huntington mouse model MRI” and classified them according to the model and MRI techniques used. Of course, the literature on these models of HD is much broader, with in particular studies involving other imaging methods such as PET. Many of these are highly complementary to the MRI studies, and provide a different perspective on these models and the disease. However, for this review, we have arbitrarily chosen to focus on MRI studies on mouse models of the disease. For complementary review on PET markers in HD, the reader can refer to a dedicated paper (Cybulska et al., 2020).

Ultimately, this review aspires to not only serve as a compendium of the current state of knowledge but also as a catalyst for further exploration. Indeed, the profusion of existing modalities and contrasts of MRI/S methods makes the design of a suitable rodent MRI/S protocol very difficult. Adding up to the diversity of HD models, the choice of the appropriate modality may be puzzling. The present review aims at giving an overview of the research in MRI/S applied to mouse models of HD and at discussing the translational potential of preclinical neuroimaging studies in HD.

## 2 Huntington’s disease

### 2.1 Symptoms

Huntington’s disease is a rare genetic neurodegenerative disease, associated with a triad of devastating symptoms (motor, cognitive and psychiatric) that rapidly affect patients’ social and family life (Walker, 2007). Motors symptoms are characterized by involuntary abnormal movements and postures (chorea, dyskinesia, and dystonia). Psychiatric disturbances include irritability, mood disorder and depression. Cognitive deficits are associated with inflexibility, perseverative behavior and impairment in strategy and planification (McColgan and Tabrizi, 2018). With disease progression, chorea disappears, and motor rigidity and dementia become more dominant. Onset of symptoms usually occurs in young individual (30–40 years of age in general). Death occurs 10 to 15 years later after a worsening of symptoms.

### 2.2 Genetics

Incidence is 1/10,000 in Europe and US, which represents approximately 40,000 patients in each region. HD is dominantly inherited and caused by a unique mutation; an abnormal CAG trinucleotide repeat expansion in the Huntingtin (HTT) gene. In addition to Huntington disease (HD), similar CAG triplet expansions in coding regions of unrelated genes exist in other rare genetic brain disorders including 6 different forms of spinocerebellar ataxias (SCA1, 2, 3, 6, 7, and 17), dentatorubral-pallidoluysian atrophy (DRPLA), and spinal and bulbar muscular atrophy (SBMA) (Bunting et al., 2022). In normal individuals, the number of CAG repeat in the HTT gene is variable but in the range of 20 repeats with no more than 35 repeats. A CAG repeat higher than 36 leads to Huntington’s disease. The repeat size in HD is very variable and most often is in the 44 CAG range. However, repeat expansion reaches more than 60–80 CAG, in rare cases, leading to an early onset disorder with rapid progression in adolescents and children. All genetic studies showed that greater numbers of repeats are associated with earlier age of onset.

In HD, the CAG repeat expansion leads to the production of a toxic mutant HTT protein that contains an abnormally expanded polyglutamine (polyQ) tract. As in all polyQ diseases, the glutamine repeat peptide segment is prone to modify the properties and functions of the protein, as compared to the wild type form. The polyQ tract makes HTT protein prone to aggregation. In addition, it induces a change in its tertiary structure/ conformation that is thought to change its interaction with a large number of partner proteins.

### 2.3 Neuropathological findings

In HD, medium-size GABAergic neurons (MSN), also called striatal projection neurons, degenerate preferentially, with a relative preservation of striatal interneurons. Compelling evidence from studies in patients and genetic animal models at different stage of the disease, indicate that there exists a long period of subclinical brain dysfunction starting years, possibly decades, before clinical onset. For example, *in vivo* neuroimaging of mutant HTT gene carriers showed that the striatum show atrophy years before the onset of motor symptoms.

Histological studies showed that HD is also associated with an astrogliosis in the striatum, with expression of molecular markers of astrocytic reactivity such as GFAP being enhanced. Quite recently, alteration of oligodendrocytes has been searched, providing compelling evidence for structural and molecular defects of these cells in the corpus callosum, striatum and the fornix (Gabery et al., 2021).

If striatal atrophy is the most striking neuropathological finding in the brain of HD patients, other brain regions are also affected to a lesser degree, depending on the stage of the disease including the cerebral cortex (layers III, V, and VI) the internal and external pallidum, the substantia nigra pars reticulate (Brouillet et al., 1999), the hypothalamus (Gabery et al., 2010).

### 2.4 Pathogenesis: main pathways

The molecular mechanisms underlying neurotoxicity in HD, as well as dysfunctions of other cell types such as astrocytes, remain poorly understood, in particular mechanisms at very early stages of the disease. However, understanding the mechanisms of early degeneration is crucial to identify the molecular targets on which innovative therapeutic interventions could act and slow down disease progression.

In HD, as in all polyQ diseases, the glutamine repeat peptide segment is prone to modify the properties and functions of the protein, as compared to the wild type form. The polyQ tract makes HTT protein prone to aggregation. The polyQ stretch within Htt protein dramatically increases its hydrophobicity and small N-terminal fragments generated by cleavage of full length m-Htt by proteases can aggregate *in vitro* (Qin and Gu, 2004). The role of these aggregates and inclusions are unclear, but it is probable that soluble or oligomerized forms of m-Htt are the molecular species mainly associated with neurotoxicity, while aggregates correspond to a cellular compensatory response to neutralize mutant HTT toxic molecular species (Saudou et al., 1998; Kuemmerle et al., 1999; Arrasate et al., 2004).

Understanding of HTT protein biology and toxicity of its mutant form has to take into account that the protein interacts with more than 300 molecular partners (Shirasaki et al., 2012), emphasizing its probable implication in a variety of cellular processes (Bates et al., 2015).

HTT protein is preferentially a cytoplasmic protein that interacts with the cytoskeleton. In HD, mutant HTT, especially N-terminal fragments can be found within the cytoplasm and the nucleus where it likely produces a dysregulation of the expression of many genes (Sugars and Rubinsztein, 2003). The function of several transcription factors or signaling pathways regulating transcription in neurons or in other brain cells is thought to be altered in HD and this could lead to progressive decline inducing dysfunction of cortico-striatal connections (Rebec, 2018) followed by death of striatal neurons.

Other early alterations are produced by mutant HTT, including defects in axonal transport, proteasome dysfunction, increased transglutaminase activity, perturbation of calcium homeostasis and synaptic function (Gunawardena et al., 2003; Li et al., 2003; Szebenyi et al., 2003; Tang et al., 2003; Bezprozvanny and Hayden, 2004; Gauthier et al., 2004), and energy metabolism and mitochondrial functions (Liot et al., 2017; Sawant et al., 2021). Deregulation of cholesterol metabolism could also be a central aspect of HD pathogenesis, especially 24OH-cholesterol that abnormally accumulates in the HD brain (Valenza and Cattaneo, 2011; González-Guevara et al., 2020; Kacher et al., 2022).

Thus, it is conceivable that mechanisms of mHTT neurotoxicity are multifactorial (Petersén et al., 1999). It is beyond the scope of the present review to go into details of all these probably important cellular/molecular defects produced by both gain and loss of function in HD. Excellent reviews on HD pathogenesis mechanisms and the physiological role of wild type HTT function can be found elsewhere that could not all be cited above.

### 2.5 Therapeutic approaches: current research

Some medication can reduce the severity of some symptoms and suffering of patients at the early stage of the disease (e.g., atypical neuroleptics). Meta-analyses of clinical trials underline that evidence for the efficacy of symptomatic treatment needs further assessment. To date, only deutetrabenazine, inhibitor of the vesicular monoamine transporter (VMAT2), produces significant beneficial effects on chorea and dystonia without inducing a disease-modifying effect in HD gene carriers (Ferreira et al., 2022). However, there is at present no efficient treatment to slow disease progression. Thus, there is still an urgent need to find efficacious therapies that could slow disease progression.

The first approach is the direct targeting of the mutant HTT gene to reduce or block the production of the mutant protein. It is known from genetic experiment in animal models that the detrimental phenotype produced by mutant HTT or mutant HTT fragment is substantially reduced if the expression of the abnormal protein is conditionally blocked in young and even adult animal (Yamamoto et al., 2000; Martín-Aparicio et al., 2001).

Then a major research effort to find new ways to block mutant HTT production or to increase its degradation took place (Tabrizi et al., 2022). The most promising approach was using antisense oligonucleotides (ASO) technologies to target the huntingtin mRNA. Several academic and biotech laboratories developed efficient ASO, tested them in animal models for toxicity and efficacy and moved rapidly toward clinical trials in symptomatic HD patients. In parallel other approaches, were developed (Tabrizi et al., 2022), including shRNA strategies stably delivered into the striatum using viral vectors-mediated gene-transfer (Thomson et al., 2022). Since targeting both normal and mutant HTT alleles might be toxic, considering crucial physiological role of HTT and the fact that a majority of HD gene carriers are heterozygous, new approaches aims at optimizing ASO and other tools to specifically target the mutant allele, and preserving the wild type HTT gene. At the preclinical stage, in mice, rats and relatively big animals like mini-pig, those strategies were markedly improved within the last years and may be tested in clinical trial in a near future assuming toxicology, safety, ethical and regulatory issues are satisfactorily addressed.

On the other hand, directly targeting the HTT gene (mutant and/or wild type) might not be the only strategy. A multitude of convincing studies in cell system and mouse models show that it is possible to markedly attenuate the HD phenotype using different pharmacological, molecular or environmental approaches acting on cellular/molecular pathways that are found to be altered in HD patients and HD models. Beyond HTT-lowering strategy, excellent and comprehensive reviews on these multiple approaches can be found (Beal and Ferrante, 2004; Dickey and La Spada, 2018). The difficulty to obtain a disease-modifying treatment may also be due to the large size of the cohorts needed for clinical trials on early-phase HD patients or presymptomatic gene carriers. To address this issue, recent development in neuroimaging, in particular with MRI/S, have provided novel *in vivo* biomarkers.

## 3 MRI-MRS: a wide variety of methods to investigate the brain

In this section, we will introduce the theoretical concepts of MRI and MRS modalities that have already been used in previous preclinical studies to measure parameters involved in the disease and that can be used as relevant biomarkers to better understand HD physiopathology and to evaluate therapeutic efficiency of future treatments. Table 1 recapitulates all modalities already used to characterize mouse models of HD.

**Table 1:** Summary of MRI/S modalities used for neuroimaging in Huntington’s disease.

Interest	Modality	Technique	Description
Morphological modifications	Anatomical imaging	Manual segmentation	Manual delineation of structures to extract volumes from high-resolution anatomical images
		Atlas-based segmentation	Image coregistration with an atlas for automated extraction of structures volumes
		Voxel-based morphometry	Voxel-wise comparison of two groups to identify clusters of voxels with significant atrophy
Metabolic modifications	^1^H-MRS	Metabolites quantification	Quantification of peak of glutamate (Glu), glutamine (Gln), N-acetyl-aspartate (NAA), myo-inositol (Ins), acetylcholine (Cho), γ-aminobutyric acid (GABA), lactate (Lac), taurine (Tau), and creatine (Cr)
	^31^P-MRS	Metabolites quantification	Quantification of peak of adenosine diphosphate (ADP), adenosine triphosphate (ATP), phosphocreatine (PCr), inorganic phosphatase (iP)
	^17^O-MRI	Metabolites quantification	Dynamic imaging of the signal after inhalation of 17O to calculate the cerebral metabolic rate of oxygen utilization (CMR02)
	CEST-MRI	gluCEST	Imaging of the CEST contrast of glutamate
Microstructural modifications	Diffusion MRI	Diffusion weighted imaging	Calculation of the Apparent Diffusion Coefficient (ADC) from the attenuation of signal from different b-values
		Diffusion tensor imaging	Derivation of Fractional Anisotropy (FA), Axial Diffusivity (AD), Radial Diffusivity (RD), from the diffusion tensor calculated from diffusion weighted experiments in different directions
		Continuous time random walk	Random Walk Model of Diffusion to calculate Dαβ and α parameters, corresponding to the tortuosity and axonal density
		Neurite orientation dispersion and density imaging	Use of multiple b-values and directions to obtain parameters of cell organization such as orientation dispersion index (ODI) and intracellular volume fraction (ICVF)
		Fixel-based analysis	Use the main orientation direction in each voxel to reconstruct white matter tracts and extract metrics such as cross-section FC or fiber density (FD)
	Magnetization Transfer imaging	MT imaging	Imaging of the MT contrast of macromolecules including myelin
		Quantitative MT	Quantitatively estimate the macromolecular proton fraction (MMPF) with high specificity to myelin
Functional modifications	Blood Oxygen Level Dependent MRI	task-based fMRI	Dynamic imaging of the BOLD contrast in response to a stimulus
		resting-state fMRI	Dynamic imaging of the BOLD contrast without any stimulus to calculate the functional connectivity (FC) between regions
Vascular modifications	Perfusion MRI	Dynamic susceptibility contrast	Dynamic imaging of the blood contrast after injection of a contrast agent to extract metrics such as the cerebral blood flow (CBF) or volume (CBV)
		3D-MR angiography	Extract micro-vessel density with a T2-weighted image following iron oxide particle injection
		Arterial spin labeling	Tagging of the blood water magnetization in a labeling plane to extract perfusion metrics without any contrast agent
		Flow-sensitive alternating inversion recovery	Variant of ASL with improved label efficiency and reduced SAR
		iVASO	Nulling of blood signal in a different plan than the imaging slice, may be sensitive to arterial CBV

### 3.1 Morphological modifications

The best-known and most widely used application of MRI are anatomical images of the brain based on T1 or T2-weighted contrasts. Segmentation of the brain in different regions of interest from high-resolution anatomical images can be performed to extract volumes, which then can be compared between a pathologic and a control group to evaluate potential atrophies. Volumetric analysis is the most commonly used method to assess disease progression. Indeed, striatal atrophy is the best *in vivo* biomarker to date to monitor the disease. However, while the acquisition of high-resolution anatomical images is available on all MRI systems, there is no consensus on image analysis strategy.

**Figure 1:**
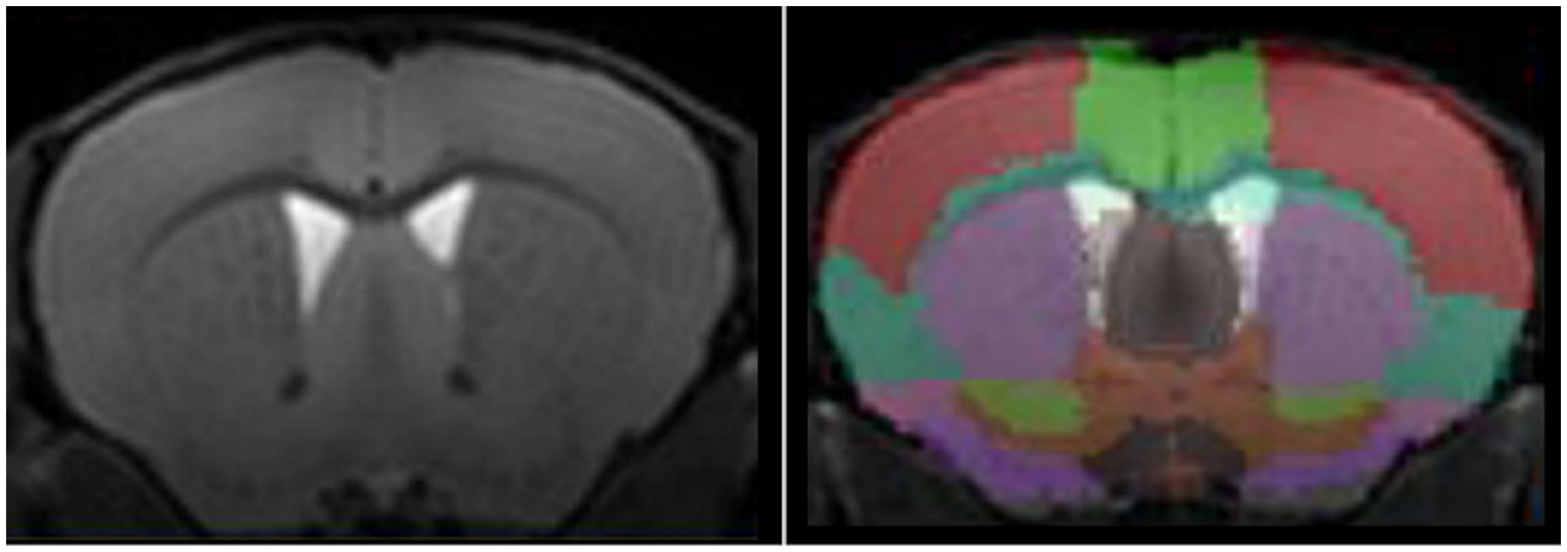
High-resolution T^1^-w image of a mouse brain acquired at 11.7 T (left panel) and automated segmentation of mouse brain using an atlas-based approach (right panel).

Automated segmentation (Figure 1) tends to be preferred to manual segmentation, due to reduction of experimenter bias. Nonetheless, different approaches are available, voxel-based, surface-based, atlas-based, or using deep learning. For detailed presentation of these techniques, the reader can refer to comprehensives reviews on the matter (Helms, 2016; Akkus et al., 2017). In brief, image voxels can be labeled either as grey matter, white matter, or corticospinal fluid, either considering voxel-by-voxel or as surfaces, at the interface between two regions. A labeled image, used as a brain atlas, can then be co-registered to the image of interest for extraction of structure volumes.

### 3.2 Metabolic modifications

#### 3.2.1 ^1^H-MRS

**Figure 2:**
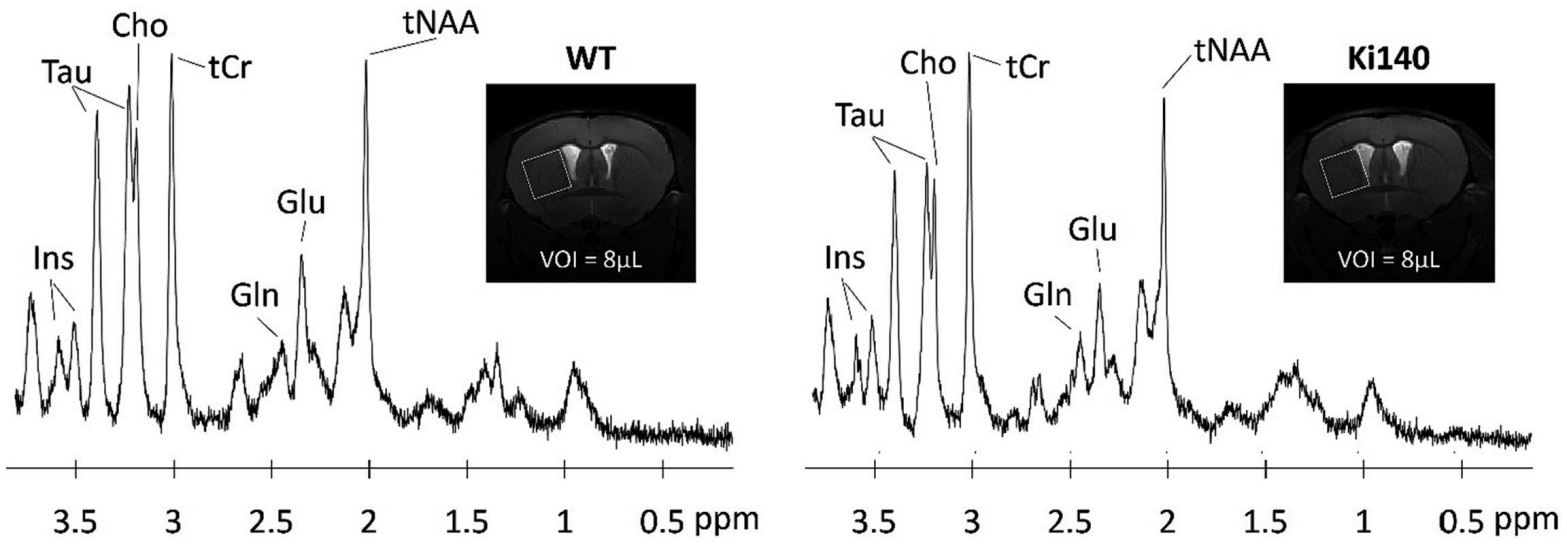
Examples of ^1^H-spectrum acquired at 11.7 T in a voxel of 8 μL located in the left striatum of WT and Ki140 mice. Inset: coronal image centered on mouse striatum with the VOI delimited by the white box. The following metabolites, total choline (tCho), total creatine (tCr), glutamate (Glu), glutamine (Gln), myo-inositol (Ins), total N-acetyl-aspartate + N-acetyl-aspartyl-glutamate (tNAA), and taurine (Tau), were reliably quantified. Adapted from Pépin et al. (2020).

Neurodegenerative diseases are often characterized by alterations of the brain metabolism, due to adaptations of the cells to maintain homeostasis despite the cascade of processes leading to degeneration. Magnetic resonance spectroscopy (MRS) is able to give insights into the metabolism of cerebral cells. By acquiring signal in a single voxel, and suppressing the free water signal, ^1^H-MRS is able to detect signal from the protons of metabolites based on their resonance frequency (Figure 2). ^1^H-MRS is thus an attractive modality for studying neurodegenerative diseases. The ability to measure the various metabolites with good accuracy depends largely on the magnetic field, but the following metabolites are classically observable on most MRI scanners: creatine (Cr), N-acetyl-aspartate (NAA), glutamate (Glu), glutamine (Gln), choline (Cho), myo-inositol (Ins), γ-aminobutyric acid (GABA), and lactate (Lac). With a voxel of acquisition placed in a brain structure of interest, e.g., caudate-putamen in HD, this modality can inform on the neurodegenerative process or glial metabolic alterations. Indeed, while a decrease of NAA will be linked to a neuronal loss, a change in the Gln/Glu ratio or in the concentration of Cho and Ins can be induced by astrocytic reactivity, as these two metabolites can primarily be found in astrocytes.

#### 3.2.2 ^31^P-MRS

By using ^31^P-MRS, one can identify and measure the predominant phosphorylated metabolites. This is especially valuable for evaluating energy metabolism, as alterations are thought to have a crucial impact on HD. This technique is particularly interesting for the measurement of phosphorylated metabolites, such as Adenosine diphosphate (ADP), adenosine triphosphate (ATP), and phosphocreatine signals, three molecules at the origin of energetic metabolism. The activity of the ATP-synthase or creatine kinases can also be derived from these metabolites. Another application is the precise measurement of the intracellular pH from the resonance frequency peak of the inorganic phosphatase (Petroff and Prichard, 1983). Hence, it is possible to get information on the cellular activity in the brain, which can be modified in neurodegenerative diseases.

#### 3.2.3 ^17^O-MRI

The 17O isotope of oxygen is detectable using MRI and is of high interest for metabolic studies (Atkinson and Thulborn, 2010). Indeed, while 17O is not visible with *in vivo* NMR, after inhalation of 17O-enriched air, increase in ^17^O-MRI signal will come from H217O produced from metabolic reactions. Thus, from repeated acquisitions of ^17^O-MRI with high temporal resolution before, during and after inhalation of 17O2, it is possible to model the cerebral metabolic rate of oxygen utilization (CMRO2) (Zhu et al., 2005). CMRO2can be impaired in neurodegenerative diseases, as it reflects the mitochondrial function. ^17^O-MRI is thus a valuable technique for metabolic biomarkers, however, its use is still limited by its cost.

#### 3.2.4 Chemical exchange saturation transfer

**Figure 3:**
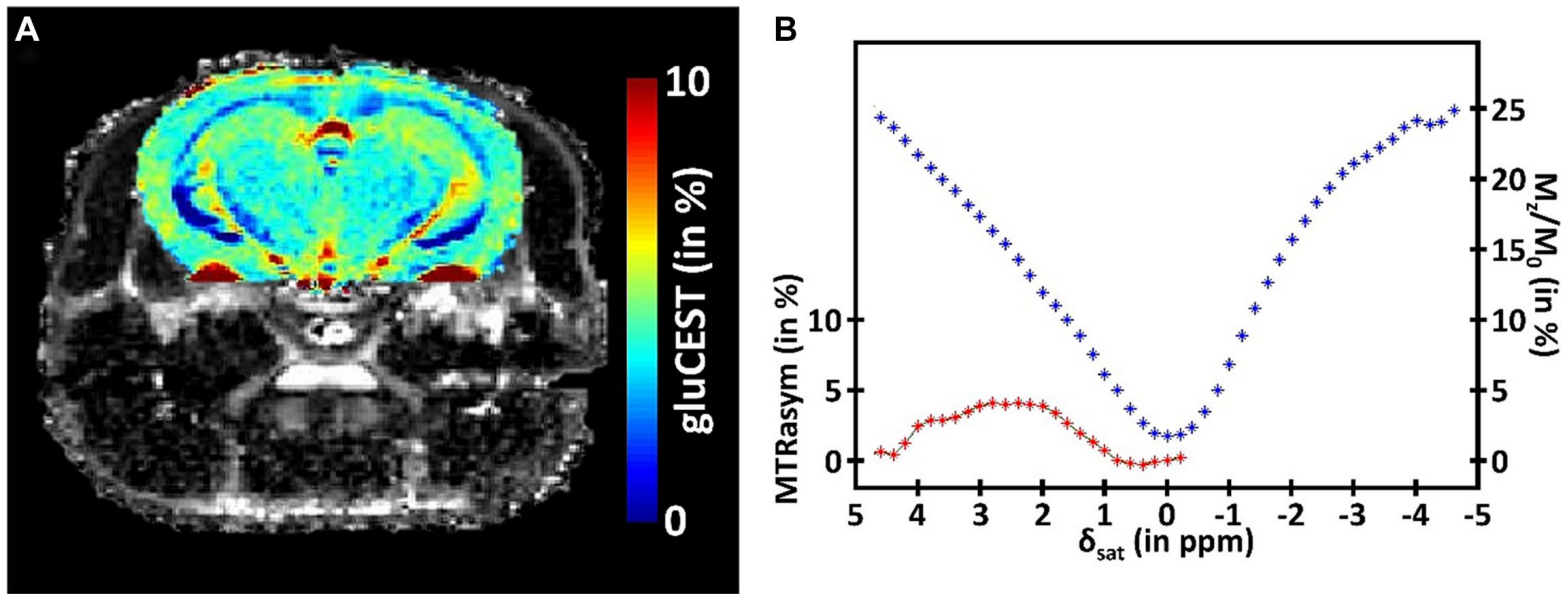
**(A)** Example of a CEST image of glutamate (gluCEST) acquired in the mouse brain at 11.7 T. **(B)** Illustration of the CEST principle. The signal of the free water is plotted in function of the frequency offset of the saturation (Zspectrum, blue curve). The specific CEST effect of a given molecule is calculated by subtracting the magnetization at a saturation frequency from the magnetization at the opposite frequency (MTRasym, red curve).

While providing valuable information about the metabolism of a specific structure, MRS suffers from its inherently low sensitivity. Consequently, this method requires acquisition of signal in large voxels that strongly limit the spatial resolution. To overtake this limitation, CEST imaging has been proposed to exploit the saturation transfer phenomenon to increase this sensitivity and be able to indirectly image some metabolites of interest (Ward et al., 2000). In short, a saturation pulse is applied at the metabolite resonance frequency, prior to the detection. This saturation is then transferred to free water, and generates reduction in the free water signal, with several magnitude orders increased sensitivity, granting the possibility to obtain a signal proportional to metabolite concentration with good resolution. In a CEST protocol, several images are acquired with saturation pulse applied at different frequency offsets. The evolution of free water signal (Mz), normalized by the reference signal (M0), and plotted as a function of saturation frequency is called the Z-spectrum (Figure 3B, blue curve). In order to isolate the specific CEST effect of a given molecule, a CEST asymmetric ratio (MTRasym) is calculated by subtracting the magnetization at a saturation frequency Mz (δsat) from the magnetization at the opposite frequency Mz (−δsat) normalized by M0: MTRasym = 100 × (Mz (−δsat) – Mz (δsat))/M0 (Figure 3B, red curve).

A range of metabolites are accessible via CEST imaging. They can be divided into different classes, depending on the type of chemical group they are composed of. Most of the molecules detectable with CEST imaging thus contain an amide (-NH), amine (-NH2), or hydroxyl (-OH) group. The first is used for the amide proton transfer (APT) technique, which is sensitive to tissue pH. While the main applications of APT are tumor and ischemia imaging, it can also be used in neurodegenerative diseases. CEST imaging of the glutamate (gluCEST, Figure 3A) is the main application of CEST imaging of the amine class, due to natural abundance of this metabolite, and can be of great interest for metabolic imaging of neurodegenerative diseases. Finally, hydroxyl groups allow to use CEST imaging to detect carbohydrates such as glucose or glycogen. Their interest is evident in a pathologic context, yet some developments are still needed to fully characterize the CEST signal from these complex molecules before using them in a clinical study.

### 3.3 Microstructural modifications

#### 3.3.1 Diffusion tensor imaging

By adding a gradient in a specific direction, symmetrically on both sides of the refocusing pulse, diffusion-weighted sequences observe a reduction of signal that is related to the diffusion of water molecules, and thus to their environment at a microscopic scale. In fact, the scale of diffusion to which the sequence is sensitive is defined by the b-value, a coefficient depending on the diffusion time and gradients amplitude. Diffusion parameters as measured with diffusion MRI can thus inform on the tortuosity of the extracellular space, the cell density, and cell shapes. However, for that purpose, data from diffusion MRI need to be modeled with a variety of models with different degrees of complexity. The most common model is that of the diffusion tensor. Diffusion Tensor Imaging (DTI) extracts the eigenvalues of the diffusion tensor based on diffusion experiments acquired in multiple directions. From eigenvalues, multiple parameters can be derived such as axial diffusivity (AD), radial diffusivity (RD), apparent diffusion coefficient (ADC) or fractional anisotropy (FA). The latter describes the degree of anisotropy inside a voxel and is the most commonly used when studying neurodegenerative diseases. Indeed, neurodegeneration, neuronal shrinkage, or demyelination, as often observed in these diseases, tend to decrease FA in the white matter, due to decreased anisotropy, while increasing FA in the grey matter. AD and RD are affected in these pathologies due to demyelination and axonal degeneration. However, this model does not take into account partial effects, and from DTI it’s impossible to disentangle the contribution from, e.g., demyelination and neuronal death. Other methods exist that propose to address this issue using multiple b-values and a more complex model, such as NODDI. For more details about advanced models, the reader is referred to relative reviews (Lakhani et al., 2020; Afzali et al., 2021). Tract reconstruction methods also exist and can be used to measure diffusion parameters along a tract of interest, or to evaluate neuroconnectivity within and between cerebral structures (Gatto et al., 2021; Gatto and Weissmann, 2022).

#### 3.3.2 Magnetization transfer

Similarly to CEST imaging, Magnetization Transfer (MT) is performed by adding a saturation pulse at a specific frequency corresponding to the resonance of protons linked to macromolecules (Wolff and Balaban, 1989). While not specific to myelin, MT has been widely used to image white matter integrity, as myelin is very abundant in these regions. Demyelination processes as observed in numerous neurodegenerative diseases result in a decreased ratio between MT-weighted and control images. Recent developments have proposed to increase the specificity of the MT technique with quantitative estimation of the macromolecular proton fraction (MMPF), such as the single-point quantitative MT (Yarnykh, 2012) or the inhomogeneous MT method (Varma et al., 2015).

### 3.4 Functional modifications

#### 3.4.1 BOLD imaging

Neuronal activation in a precise cortical region leads to local increase of the blood flow, which locally reduces the deoxyhemoglobin/oxyhemoglobin ratio. As deoxyhemoglobin is highly paramagnetic while oxyhemoglobin is diamagnetic, neuronal activation is accompanied with a local increase of the so-called “Blood Oxygen Level Dependent” (BOLD) signal, that is at the foundation of functional MRI (Forster et al., 1998).

This effect can be used in the study of neurodegenerative diseases by measuring the cortical activation in response to a stimulus (task-based) or without any stimulus (resting-state). In a task-based fMRI experiment, the subject is submitted to a stimulus, such as a visual stimulation or an odor, while images are acquired with high temporal resolution to observe the variation of BOLD signal in a region of interest associated with the stimulus.

#### 3.4.2 Resting state fMRI

Catching increasing interest over the last few years, the resting-state fMRI (rs-fMRI) technique proposes to observe spontaneous fluctuations of BOLD signal in the brain, without any stimulus. The correlations between fluctuations in the signal in voxels from different brain then can inform about the functional connectivity between those regions (Barkhof et al., 2014).

From rs-fMRI data, it is possible to measure functional connectivity (FC) between regions of interests given by an atlas, or to extract regions of coherent activation directly from the data using independent component analysis (ICA) or dictionary learning analysis. Recently, dynamic ICA has been proposed to generalize this research of correlation with the postulate that brain organization in functional networks is not static and one region can be transiently part of several networks (Allen et al., 2014).

Based on FC matrix, graph theory is another tool for rs-fMRI data analysis (Wang et al., 2010). A spatialization algorithm is used to represent the regions of the brain on a graph, with FC values weighting the links between regions. From the final graph, several measures have been proposed as biomarkers of the organization of the network, such as the shortest path length (SPL) or degree of a node.

### 3.5 Vascular modifications

#### 3.5.1 Dynamic susceptibility imaging

A lot of techniques have been developed to examine brain vascular integrity using MRI. Dynamic susceptibility imaging (DSC) is based on the injection of gadolinium in the blood. The induced change in blood susceptibility leads to a signal loss, first visible in large arteries and then in the whole brain as gadolinium gets to small vessels (Copen et al., 2011). Perfusion metrics, such as Cerebral Blood Flow (CBF) or Cerebral Blood Volume (CBV), which can be impacted in neurodegenerative diseases, can then be calculated from the subtraction between imaging before and after injection (Jezzard et al., 2018).

#### 3.5.2 Arterial spin labeling

In order to avoid the injection of an exogenous contrast agent, Arterial Spin Labeling (ASL) has also been proposed for perfusion imaging. This technique relies on the tagging of the blood water magnetization in a labeling plane. The acquired brain image after tagging is compared to the same acquisition without inversion to extract CBF and CBV. ASL principle has been declined in several sequences such as pseudo-continuous ASL (pCASL) (Hirschler et al., 2018), which is presently the favored labeling approach, or flow-sensitive alternating inversion recovery pulsed ASL (FAIR), which displays improved labeling efficiency and reduced SAR.

#### 3.5.3 Other techniques of perfusion MRI

A variant of fMRI, referred to as gadolinium enhanced imaging, has also been proposed for evaluation of a relative CBV (rCBV) (Moreno et al., 2007). While this technique is not fully quantitative compared with DSC imaging, it is based on intraperitoneal injection of gadolinium rather than intravenous, and thus is easier to perform longitudinally.

MR angiography (MRA) has been proposed to visualize the micro vascularity and extract microvessel density with a T2-weighted image following iron oxide particle injection (Lin et al., 2009).

Finally, inflow vascular-space-occupancy (iVASO) MRI has been recently developed as an improvement of VASO (Hua et al., 2011). The latest technic is used to assess alterations in cerebral blood volume by nulling the blood signal, but suffers from poor SNR due to non-selective inversion pulse. In contrast, iVASO proposes to null the signal in a different plan that the imaging slice, thus improving SNR. Furthermore, the method allows to be sensitive to arterial CBV only by tunning the inversion time and slice gap between inversion and imaging slices.

## 4 MRI and MRS findings in genetic mouse models of HD

In this section, we will present main mouse models developed to mimics different stages of HD pathology. We will also try to provide an extensive review of the relevant biomarkers already studied in the literature to characterize these animal models.

### 4.1 Transgenic models of HD

In transgenic models, a fragment or the entire *HTT* gene is inserted randomly into the murine genome. Such models display rapid and severe phenotypes with reduced lifespan.

#### 4.1.1 R6/2

The R6/2 line is a widely used model of HD, expressing a N-terminal fragment of HTT, with 144 to 150 CAG repeats (Mangiarini et al., 1996). They show a very severe phenotype with early symptoms and short lifespan of 10–13 weeks (Menalled and Chesselet, 2002; Jenkins et al., 2005). R6/2 mice present abnormal Rotarod performance as early as 4 weeks and hypokinesia at 6 weeks (Stack et al., 2005; Menalled et al., 2009). Cognitive symptoms, such as altered spatial learning, were also observed in young animals (Lione et al., 1999).

Structural MRI studies have shown very early striatal atrophy (−30%) at 4–5 weeks, followed with atrophy of the neocortex (−10%), and hippocampus (−32%), and whole brain atrophy (−19%) (Sawiak et al., 2009a; Zhang et al., 2010; Cepeda-Prado et al., 2012; Parievsky et al., 2012; Lin et al., 2013; Tereshchenko et al., 2019). Early atrophy (3 weeks) has also been reported in the piriform cortex (Aggarwal et al., 2012) as well as the corpus callosum at 8 weeks (Rattray et al., 2013). Clusters of voxels showing significant atrophy were reported in the cortex, cerebellum, basal ganglia, hippocampus, thalamus and hypothalamus (Sawiak et al., 2009b). Several studies have reported hypertrophies of ventricles and globus pallidus (Ferrante et al., 2002; Sawiak et al., 2009a; Zhang et al., 2010).

^1^H-MRS studies reported a decrease of the total N-acetyl-aspartate (tNAA) concentration in striatum and cortex of R6/2 mice, starting at 4 weeks associated with striatal atrophy (Jenkins et al., 2000, 2005; Tsang et al., 2006; Tkac et al., 2007; Zacharoff et al., 2012). Taurine (Tau) and glutamine (Gln) were abnormally elevated at 8 weeks (Jenkins et al., 2000; Tkac et al., 2007; Zacharoff et al., 2012), while conflicting reports on glutamate (Glu), observed either downregulated (Jenkins et al., 2000) or upregulated (Tkac et al., 2007), may reflect homeostasis imbalance in this animal model. Total creatine (tCr) was also reported to be increased in this model. In addition, CMRO2 measured with ^17^O-MRI was significantly reduced when challenged with a drug mimicking ATP generation blockade (Lou et al., 2016).

Apparent Diffusion Coefficient (ADC) increased in globus pallidus at 10–12 weeks (Vorisek et al., 2017). Corpus callosum showed decreased Fractional Anisotropy (FA) values at 4 weeks (Xiang et al., 2011; Gatto et al., 2015, 2019), while Axial Diffusivity (AD), Radial Diffusivity (RD) and Mean Diffusivity (MD) were increased. Such results reveal undeniable defect of the microstructure of brain tissues. DTI was used as a biomarker of myelination to evaluate the effect of a reprogramming factor injection in R6/2 mice (Yu et al., 2021). Continuous Time Random Walk (CTRW) model on the same mice showed decreased Dαβ and α parameters, highlighting increased tortuosity and axonal density of corpus callosum (Gatto et al., 2019).

The Cerebral Blood Volume (CBV) was significantly increased in the striatum and temporal, prefrontal and motor cortices of R6/2 mice at 6 weeks, and later in hippocampus and thalamus (Cepeda-Prado et al., 2012; Parievsky et al., 2012). In addition, in spite of increased vessel volume fraction at 7 weeks (Lin et al., 2013), the fraction of reactive vessels after a challenge was decreased in the cortex and striatum, showing a loss of plasticity in these regions that remain in a permanent state of activation (Hsiao et al., 2015).

Altogether, these results describe a model of HD with a strong phenotype, showing vulnerability of the striatum that seems to imitate that of the human disease.

#### 4.1.2 R6/1

R6/1 is an early transgenic mouse model of HD, expressing the exon 1 of *HTT* with 116 CAG repeats (Mangiarini et al., 1996) under the promoter of human HTT. Memory impairments appear around 12–14 weeks (Mazarakis et al., 2005; Cayzac et al., 2011) and with lowered motor capacity is observed from 2 months (Naver et al., 2003). R6/1 mice have a decreased lifespan of 8–10 months.

Morphological analysis by MRI showed significant atrophy of the whole brain, including the striatum, cortex, and hippocampus in 17-week-old mice (Rattray et al., 2013). Cortical subregions such as retrosplenial cortex and somatosensory cortex were also strongly affected.

Metabolic biomarkers were also investigated in the striatum of R6/1 mice. A 36% decreased of tNAA level was measured at 7 months associated with a striatal atrophy (Pépin et al., 2020). Interestingly, the decrease of tNAA level and striatum volume were measured in the absence of neuronal loss at 7 weeks and can be explained by neuron shrinkage.

DTI showed reduced FA in white matter and increased FA in grey matter of R6/1 mice as early as 11 weeks (Gatto et al., 2021). These results were completed by NODDI analysis, which showed altered orientation dispersion index (ODI) and decreased intracellular volume fraction (ICVF) associated with neuroinflammation. Tract reconstruction performed at different timepoints in the R6/1 mouse brain showed reduced neuroconnectivity within grey matter structures in an early phase (Gatto and Weissmann, 2022).

R6/1 imaging thus describe a model with high interest for the study of neuronal impairment, starting by neuron shrinkage and followed by neurodegeneration leading to global atrophy. Interestingly, the striatum is particularly vulnerable in this model, similarly to the human form of the pathology.

#### 4.1.3 N171-82Q

The N171-82Q line express 171 residues of human HTT 5′-end, including 82 CAG repeats (Schilling et al., 1999). The fragments are only expressed in neurons under the murine prion promoter. Compared to control mice, N171-82Q mice present a broad spectrum of cognitive and motor symptoms and reduced lifespan to 5–6 months.

Abnormal weight loss is visible after 2 months, and motor performances as evaluated using Rotarod test are impaired at 3 months. Memory impairments were detected in 14-weeks-old mice (Ramaswamy et al., 2007).

A progressive whole brain atrophy was reported with a significant ~20% decrease reached at 10 weeks in the striatum, neocortex and thalamus (Cheng et al., 2011; Aggarwal et al., 2012; Lee et al., 2014; Zhou et al., 2018). Interestingly, white matter was affected even earlier with a 20% atrophy of corpus callosum detected at 6 weeks.

Metabolic alterations were measured with ^1^H-MRS with a − 27% decrease of tNAA level in the striatum of 4 months old mice (Jenkins et al., 2005).

Resting state fMRI was also performed in 18-weeks-old N171-82Q mice to study functional and network modifications (Li et al., 2017). While similar networks were identified in HD and WT mice, correlation strengths between different regions were decreased in HD animals. In particular, the motor and prefrontal cortex were less correlated, as well as retrosplenial cortex and thalamus. Striatum also presented a decreased connectivity with every other region excepted retrosplenial cortex. Interestingly, connectivity impairments were positively correlated to striatal atrophy.

No change in micro-vascularization was found in N171-82Q mice when compared with WT, while N171-82Q/GFAP bred mice, expressing HTT fragments in both neurons and astrocytes showed increased vascularization, showing that glial expression of HTT fragments worsens the phenotype of this model (Hsiao et al., 2015).

These results suggest that N171-82Q mice are relevant models of neuronal degeneration in HD with striatal vulnerability but may be inconsistent with some of the glial alterations reported in HD pathogenesis.

#### 4.1.4 Main characteristics of transgenic models

In general, transgenic mice models exhibit severe phenotypes and reduced lifespan. Table 2 provides a summary of imaging findings in these models. Such models can prove highly valuable for quickly assessing potential treatments or investigating mechanisms of neurodegeneration. However, it’s worth noting that the overexpression of partial fragments of the HTT protein may not result in the same neurodegenerative processes as the full-length mutant HTT protein. Additionally, the rapid progression of the disease in these models makes difficult to assess subtle changes occurring during the pre-symptomatic phase of the disease.

**Table 2:** Summary of imaging results in the transgenic mouse models of HD.

Model	Interest	Technique	Main findings	Age	References
R6/1	Morphological modifications	Manual segmentation	Whole brain atrophy	17 weeks	Rattray et al. (2013)
			Atrophy of striatum, cortex and hippocampus	17 weeks	
		VBM	Vulnerable retrosplenial and somatosensory cortices	17 weeks	
			Homogeneous atrophy of the striatum	17 weeks	
	Metabolic modifications	^1^H-MRS	tNAA decreased in striatum	7 months	Pépin et al. (2020)
	Microstructural modifications	DTI	Reduced FA in white matter	11 weeks	Gatto et al. (2021)
			Increased FA in grey matter	11 weeks	
		NODDI	Increased ODI	11 weeks	
			Decreased ICVF	11 weeks	
		Tractography	Increased cortical neuroconnectivity	11 weeks	Gatto and Weissmann (2022)
R6/2	Morphological modifications	Manual/atlas-based segmentation	Whole brain atrophy	4–5 weeks	Sawiak et al. (2009a), Zhang et al. (2010), Cepeda-Prado et al. (2012), Parievsky et al. (2012), Lin et al. (2013), Tereshchenko et al. (2019)
			Striatal atrophy	4–5 weeks	
			Cortical and hippocampal atrophy	4–5 weeks	
			Atrophy of the corpus callosum	8 weeks	Rattray et al. (2013)
			Increased atrophy rate of Piriform cortex	3–12 weeks	Aggarwal et al. (2012)
			Hypertrophy of ventricles and globus pallidus	4–5 weeks	Sawiak et al. (2009a), Zhang et al. (2010), Ferrante et al. (2002)
		VBM	Vulnerable regions in cortex, cerebellum, basal ganglia, hippocampus, thalamus, and hypothalamus	18 weeks	Sawiak et al. (2009b)
	Metabolic modifications	^1^H-MRS	tNAA decreased in striatum and cortex	4 weeks	Jenkins et al. (2005, 2000), Tkac et al. (2007), Zacharoff et al. (2012), Tsang et al. (2006)
			Increased Tau and Gln in striatum	8 weeks	Jenkins et al. (2000), Tkac et al. (2007), Zacharoff et al. (2012)
			tCr increased in striatum	8 weeks	Tkac et al. (2007)
		^31^P-MRS	No difference in ATP, ADP, or ATP production rate	6–13 weeks	Menalled et al. (2003)
		^17^O-MRI	Reduction of CMRO2 when challenged	9 weeks	Lou et al. (2016)
	Microstructural modifications	DWI	Increased ADC in globus pallidus	10–12 weeks	Vorisek et al. (2017)
		DTI	Decreased FA in corpus callosum	4 weeks	Gatto et al. (2015, 2019), Xiang et al. (2011)
			Increase of AD, RD, MD in corpus callosum	4 weeks	
		CTRW	Decreased Dαβ and α in corpus callosum	60 days	Gatto et al. (2019)
	Vascular modifications	DSC	Increased CBV and CBF in striatum and cortex	6 weeks	Cepeda-Prado et al. (2012), Parievsky et al. (2012)
		BOLD	Decreased fraction of reactive vessels	7 weeks	Hsiao et al. (2015)
		FAIR	Decreased CBF reaction to carbogen challenge	7 weeks	
N171-82Q	Morphological modifications	Manual/atlas-based segmentation	Whole brain atrophy	10 weeks	Aggarwal et al. (2012), Cheng et al. (2011), Lee et al. (2014), Zhou et al. (2018)
			Atrophy of the striatum, cortex, thalamus	10 weeks	
			Atrophy of the corpus callosum	6 weeks	
	Metabolic modifications	^1^H-MRS	Decreased tNAA in the striatum	4 months	Jenkins et al. (2005)
	Functional modifications	rs-fMRI	Decreased FC between striatum and other regions	18 weeks	Li et al. (2017)
			Decreased FC between motor and prefrontal cortex	18 weeks	
			Decreased FC between retrosplenial cortex and thalamus	18 weeks	
	Vascular modifications	3D-MRA	Increased vascularization in N171-82Q/GFAP mice	16–18 weeks	Hsiao et al. (2015)

### 4.2 Models expressing full-length human HTT with artificial chromosome

In order to express the full-length human HTT, other models were generated that express mutant HTT via an artificial chromosome from yeast (YAC) or bacteria (BAC). These models have the advantage to allow expression of the full-length human HTT under its own promoter, which is of high interest for studying the behavior of full-length mutant or wild-type HTT, especially for evaluation of gene therapy targeting gene regions other than exon 1.

#### 4.2.1 YAC128

YAC128 mice express full-length human HTT with 128 CAG repeats, inserted in aa yeast artificial chromosome construct under the control of the human HTT promoter (Slow et al., 2003). These mice present normal lifespan and, in contrast with other models, increased body weight after 32 weeks. They show mild motor symptoms such as reduced Rotarod performance starting around 4 months and mild and late hypokinesia (van Raamsdonk et al., 2005; Menalled et al., 2009; Brooks et al., 2012). These defects were accompanied by motor-learning deficit highlighted with reduced accuracy in serial implicit learning task (Brooks et al., 2012).

Imaging studies showed consistent morphological phenotype, with atrophy of the striatum (−3.5%) occurring at 3 months, followed by atrophy of the piriform and motor cortices, thalamus, olfactory bulbs and corpus callosum (Lerch et al., 2008a, b; Carroll et al., 2011a,b; Lewandowski et al., 2013; Garcia-Miralles et al., 2016; Petrella et al., 2018). Though, some regions including somatosensory cortex, septum and frontal cortex are reported to increase in volume relatively to whole brain (Lerch et al., 2008a, b).

YAC128 mice display metabolic profiles measured by ^1^H-MRS in apparent contradiction with other models. Their metabolic profile showed increase of tNAA and total choline (tCho) levels at 6 months, in contrast with all the previously cited models, followed by decrease at 12 months (Petrella et al., 2018). tCr were also increased at all timepoints in YAC128 mice, similarly to what was observed in R6/2 at early stage.

A decrease of FA value was measured in the corpus callosum as early as 1.5 month (Garcia-Miralles et al., 2016; Teo et al., 2016). Surprisingly, FA decrease was also observed in grey matter (Petrella et al., 2018), in contradiction with other models. One possible explanation is an impairment in oligodendrocytes capacity to form healthy myelin sheets.

In addition, resting-state fMRI showed reduced functional connectivity between the regions constitutive of the Default Mode Network (Chang et al., 2018). Noticeably, the amplitude of low-frequency fluctuations was increased in HD mice, probably to compensate the lack of connectivity between regions.

Longitudinal DSC study on YAC128 mouse brain showed an accelerated reduction of rCBV over time in the dorsolateral striatum, contrasting with results in other models (Lewandowski et al., 2013).

#### 4.2.2 Main characteristics of models expressing full-length human HTT with artificial chromosome

As previously mentioned, another model expressing mutant HTT via an artificial chromosome from bacteria has also been generated (BACHD). This model has been widely used to study pathogenesis of HD and to establish proof of concept for preclinical trials. Except one study showing a ~ 10% atrophy of the striatum at 15 months (Jiang et al., 2011), no further MRI/S characterization has been performed previously. Thus, we did not devote a specific section in this review for this model.

Nonetheless, such result suggests a very slow progression of neurodegeneration. Main findings were summarized in Table 3. High tNAA levels were measured at early stage, then lower levels than WT mice at later age, suggesting brain compensatory mechanisms that take place in young animals to slower the disease progression. In addition, low-frequency fluctuations as measured with rs-fMRI also support the idea of a compensation of neuronal dysfunction.

**Table 3:** Summary of imaging results in the YAC128 mouse model.

Model	Interest	Technique	Main findings	Age	References
YAC128	Morphological modifications	Manual segmentation	Atrophy of the striatum, prefrontal cortex, globus pallidus, and hippocampus	3–12 months	Lewandowski et al. (2013), Petrella et al. (2018)
		Automated Segmentation	Whole brain atrophy	3 months	Carroll et al. (2011a, b)
			Striatal atrophy	3 months	
			Atrophy of the striatum, piriform cortex, retrosplenial cortex	9 months	Garcia-Miralles et al. (2016)
		VBM	Local atrophy in subregions of the striatum, thalamus, white matter, frontal cortex	8 months	Lerch et al. (2008a)
			Local hypertrophy in subregions of sensori-motor cortex, cerebellum, septum, fimbria, entorhinal cortex	8 months	
			Increased cortical thickness	8 months	Lerch et al. (2008b)
	Metabolic modifications	^1^H-MRS	tNAA increase	6 months	Petrella et al. (2018)
			tNAA decrease	12 months	
			Decreased tCho	12 months	
			Increased tCr	6–12 months	
	Microstructural modifications	DTI	Decreased FA in the corpus callosum	1.5–2 months	Garcia-Miralles et al. (2016), Teo et al. (2016)
			Normal FA in white matter	12 months	Teo et al. (2016)
			Increased MD in striatum and thalamus	3–9 months	Petrella et al. (2018)
			Increased FA longitudinal decrease in striatum, prefrontal cortex, globus pallidus, hippocampus, and thalamus	3 months	
	Functional modifications	rs-fMRI	Decreased functional connectivity, especially between striatum and somatosensory cortex	10.5 months	Chang et al. (2018)
			Increased amplitude of low-frequency fluctuations	10.5 months	
	Vascular modifications	DSC	Longitudinal acceleration of rCBV reduction	44 weeks	Lewandowski et al. (2013)

### 4.3 Knock-in models

Knock-in models of HD are based on the direct insertion of CAG repeats into the murine *htt* gene, also called *Hdh*. Thus, these models are genetically closer to endogenous HD in patients. Knock-in mouse models are characterized by a slower progression and reduced severity of symptoms as compared to exon-1-expressing models and the phenotype is easier to assess in young adults as compared to BACHD and YAC models. Knock-in mice can be bred heterozygous or homozygous, modifying phenotype severity (Menalled, 2005). The severity is also modulated by the size of the CAG expansion, and successive generations of knock-in models with 50, 71, 92, 94, 111, 140, or 188 CAG repeats have been developed. Models with a high number of CAG repeats are of high interest since they progressively develop the pathology within the first year of life and showed many similarities with early HD patients.

#### 4.3.1 HdhQ111

With 111 CAG repeats, HdhQ111 mice show a very mild phenotype with preserved lifespan (Menalled et al., 2009). Motor performances are slightly reduced using the Rotarod test in homozygous female. These mice also present early nuclear accumulation of mHtt fragments (Wheeler et al., 2000, 2002; Lloret et al., 2006) and significant reduction of body weight.

^1^H-MRS allowed to measure reduced Tau level in the striatum of 6- and 13-weeks-old mice and increased Gln level at 13 weeks. Finally, an increase of Cr and a decrease of Phosphocreatine (PCr) measured at 6 weeks were back to control levels at 13 weeks (Tkac et al., 2012). This result suggests a homeostatic response of the cells to recover normal concentrations of these metabolites.

This hypothesis was further explored by ^31^P-MRS in the cortex (Tkac et al., 2012). While PCr and ATP concentrations were similar between HdhQ111 and control mice, ADP concentration and ATP production rate were increased at 6 weeks but not at 13 weeks. These results could be explained by a homeostatic process forcing the cell to produce more ATP at early stage of the disease.

#### 4.3.2 HdhQ140

These mice show a mild progression of the disease, similar to HdhQ111. Homozygous mice present early impairments of motor functions, with hyperkinesia at 1 month, and hypokinesia at 4 months (Menalled et al., 2003). Rotarod test showed reduced performances of 4 month-old homozygous HdhQ140 mice (Hickey et al., 2008). Study on wild-type, heterozygous and homozygous showed a progression of the severity of locomotor deficits with genotype (Pépin et al., 2016). Long-term memory defect has been reported at 16 weeks (Simmons et al., 2009).

Structural MRI revealed striatal atrophy at 12 months but only significant in homozygous mice (Pépin et al., 2016). In heterozygous mice, atrophy of the striatum, and frontal, motor, and retrosplenial cortices were observed lately at 18 months (Pérot et al., 2022).

^1^H-MRS showed metabolic modifications including reduced levels of Tau and tNAA in the striatum (Pépin et al., 2016). Glu was also reduced while Gln was increased in homozygous mice. NAA decrease in the striatum was dependent on the genotype (−17% for heterozygous mice and − 24% for homozygous mice) and was significantly correlated to striatal atrophy.

To explore further glutamate impairments with a better spatial resolution, gluCEST imaging was performed on 12-months old mice (Pépin et al., 2016). The authors found a significant decrease of the gluCEST signal in several anterior structures of the brain, including corpus callosum, striatum and piriform cortex. Interestingly, corpus callosum was the most impacted structure and the only one also significantly affected in heterozygous mice. Longitudinal follow-up of heterozygous HdhQ140 mice showed that gluCEST reduction starts at 8 months in the corpus callosum, followed by frontal and piriform cortices as well as pallidum at 12 months (Pérot et al., 2022).

Longitudinal gluCEST and volumetric imaging were combined with MT imaging, showing defects in corpus callosum using Tract-Based Spatial Statistics (TBSS) analysis in heterozygous mice (Pérot et al., 2022). Clusters of voxels with reduced FA were evidenced in the anterior part of the corpus callosum. Additionally, MT signal was reduced in the septum at 12 months.

Such results highlight the potential of multiparametric, longitudinal imaging applied to knock-in mouse models of HD for better understanding of the pathogenesis mechanisms. However, due to the very mild phenotype of HdhQ140 mice, knock-in models with faster or more severe progression have been developed to allow easier observation of effect of the mutation in heterozygous mice.

#### 4.3.3 HdhQ150

The phenotype of 90-weeks-old homozygous HdhQ150 mice was comparable to the one of 12 to 14-weeks-old R6/2 mice (Woodman et al., 2007). HdhQ150 showed abnormal gait, reduced performance in Rotarod test and late tendency to clasp., with average onset of symptoms occurring around 25 weeks for homozygous and 60 weeks for heterozygous mice (Lin et al., 2001). Motor symptoms are preceded by cognitive impairments, that can be observed at 4 months (Brooks et al., 2012).

Imaging studies on homozygous HdhQ150 showed morphological modifications, including whole brain atrophy with significant onset at 36 weeks (Rattray et al., 2017). Striatum and cortex were the first to display significant atrophy at 15 weeks followed by hippocampal atrophy at 35 weeks. Corpus callosum was also significantly atrophied at 12 months.

#### 4.3.4 zQ175

The zQ175 mice are derived from HdhQ140 model with approximately 190 CAG. Homozygous zQ175 show reduced body weight starting at 5 months, and slightly reduced lifespan (90 weeks). They show significant motor abnormalities at 4.5–6 months of age (Menalled et al., 2012; Peng et al., 2016; Kacher et al., 2019; Wang et al., 2021).Two-choice swim tank test revealed procedural memory deficit in 10 to 12-months old homozygous mice (Heikkinen et al., 2012). The original zQ175 model has a floxed neomycin resistance cassette (neo cassette) upstream of the Htt gene locus. The neo cassette may reduce Hdh gene expression in the context of the long CAG repeat, which might explain why the phenotype in zQ175 is not markedly more pronounced than in HdhQ140 mice (Southwell et al., 2016; Wu et al., 2022).

zQ175 mice present strong morphological modifications, as revealed by structural MRI. In homozygous mice, striatal (−13%), cortical (−9%) and whole brain (−6%) atrophy were significant at 3 months (Heikkinen et al., 2012; Peng et al., 2016; Bertoglio et al., 2018). In heterozygous, striatal and cortical atrophies were reported at 4 months followed by global atrophy of the brain at 12 months (Tereshchenko et al., 2019).

In consistence with other knock-in models, NAA (−23%) and Glu (−14%) levels were decreased in the striatum of homozygous zQ175 mice at 4 months (Heikkinen et al., 2012; Peng et al., 2016). Interestingly, Glu level seems to be restored at 9 months, while Gln level was increased. Finally, at 12 months Glu level was decreased again, associated with decrease of NAA and GABA levels, while Gln level remained elevated. Such metabolic fluctuations highlight homeostatic response that can occur in the brain of HD animals.

Functional MRI was also performed on awake zQ175 mice challenged with almond odor (Ferris et al., 2014). Activation response to almond odor was significantly reduced in frontal cortex, dentate gyrus and subiculum of both heterozygous and homozygous mice and in hypothalamus and olfactory bulbs of homozygous mice only. This result demonstrates the potential of fMRI to analyze functions of the brain such as odor recognition as biomarker of the disease.

Finally, vascular modifications were recently assessed in the zQ175 model using iVASO MRI (Liu et al., 2021). Arteriolar cerebral blood volume in the striatum of heterozygous zQ175 mice followed an abnormal trajectory. CBVa values were superior to WT mice at 3 months and followed by progressive decline, while control mice remain stable, leading to inferior values in zQ175 at 9 months. The vascular impairment was confirmed by histological study and CBVa values were successfully used as a biomarker for evaluation of a gene silencing therapy.

#### 4.3.5 Main characteristics of knock-in models

Table 4 summarizes MRI/S findings in knock-in models of HD. In general, knock-in models show mild morphological alterations, that can be preceded by progressive metabolic, microstructural, functional or vascular impairments. The results highlight the interest of these models for the study of presymptomatic phase of HD. In particular, longitudinal, multiparametric imaging studies bring a lot of information on the pathogenesis and could be useful for identification of therapeutics candidates.

**Table 4:** Summary of imaging results in knock-in mouse models of HD.

Model	Interest	Technique	Main findings	Age	References
HdhQ111	Metabolic modifications	^1^H-MRS	Decreased Tau in striatum	6–13 weeks	Tkac et al. (2012)
			Increased Cr/PCr	6 weeks	
			Normal Cr/PCr	13 weeks	
		^31^P-MRS	ADP and ATP production rate increased	6 weeks	
			Normal ADP and ATP production rate	13 weeks	
HdhQ140	Morphological modifications	Manual segmentation	Atrophy of the striatum in homozygous mice	12 months	Pépin et al. (2016)
		Automated segmentation	Striatal and cortical atrophy in heterozygous mice	18 months	Pérot et al. (2022)
	Metabolic modifications	^1^H-MRS	Decreased tNAA, Glu and Tau in the striatum	12 months	Pépin et al. (2016)
			Gln increase in homozygous	12 months	
		gluCEST	GluCEST reduction in the corpus callosum	8 months	Pépin et al. (2016), Pérot et al. (2022)
			GluCEST reduction in the cortex, pallidum and in the striatum in homozygous	12 months	
	Microstructural modifications	MT	Reduced MT in the septum	12 months	Pérot et al. (2022)
		DTI	Clusters of reduced FA in anterior part of corpus callosum	5 months	
HdhQ150	Morphological modifications	Manual segmentation	Whole brain atrophy	36 weeks	Rattray et al. (2017)
			Striatal and cortical atrophy in homozygous	15 weeks	
			Atrophy of the corpus callosum	12 months	
		VBM	Atrophied regions in the motor and somato-sensory cortices, striatum and thalamus	15 weeks	Rattray et al. (2017)
zQ175	Morphological modifications	Manual segmentation	Whole brain atrophy	3 months	Heikkinen et al. (2012), Bertoglio et al. (2018)
			Striatal and cortical atrophy	3 months	
		Atlas-based segmentation	Atrophy of 46 brain areas in homozygous compared to heterozygous only	12 months	Ferris et al. (2014)
		VBM	Whole brain atrophy (heterozygous)	12 months	Tereshchenko et al. (2019), Peng et al. (2016)
			Atrophied regions in the striatum and cortex (heterozygous)	4 months	
	Metabolic modifications	^1^H-MRS	Reduced tNAA in the striatum (homozygous)	4 months	Peng et al. (2016), Heikkinen et al. (2012)
			Decreased Glu	4 & 12 months	
			Normal Glu	9 months	
			Increased Gln	9–12 months	
			Decreased GABA	12 months	
	Functional modifications	Task-based fMRI	Reduced activation in frontal cortex, dentate gyrus, and subiculum of heterozygous and homozygous mice	12 months	Ferris et al. (2014)
	Vascular modifications	iVASO	arteriolar CBV superior to WT in baseline, inferior to WT in heterozygous	9 months	Liu et al. (2021)

## 5 Translational MRI: from preclinical developments to clinical applications

As already shown, preclinical neuroimaging studies allowed thorough characterization of mouse models of HD and provide relevant biomarkers to precisely study different phases or pathways of the disease. However, these biomarkers cannot always be transferred in their current state to clinical studies. First, animal models do not fully replicate the human form of the disease. They cannot include as much variability in terms of genetics, medical background, and number of CAG repeats as patients. The latter being certainly the most critical aspect as the age of onset differs significantly between patients due to expansion length, while animal models show reduced variability in the disease course. The other main limitation in translating preclinical protocol to clinical studies is more a technical issue. Indeed, most of preclinical studies are performed on high field MRI scanners so the sensitivity of the different MRI/S methods will be reduced at clinical fields. In addition, clinical protocols will have to cope with additional constrains such as SAR limitation, reduced scan time, movement artifacts or downgraded spatial resolution so their applications will therefore be greatly reduced.

However, despite these limitations, number of studies have already demonstrated the importance of MRI/S for the study of HD in a clinical context. The present section will recapitulate the main results obtained in HD patients using clinical MRI, and how preclinical studies could help to find relevant biomarkers of the disease.

### 5.1 Morphological modifications in HD patients

#### 5.1.1 Striatal atrophy

Striatal atrophy is the most reported marker of Huntington’s disease as measured by MRI (Jernigan et al., 1991; Harris et al., 1992; van den Bogaard et al., 2012; Werner et al., 2014; Wolf et al., 2014). Striatal atrophy can be measured before onset of motor symptoms and is progressive from presymptomatic to symptomatic phase (Tabrizi et al., 2009; Paulsen et al., 2010). This makes striatal volume a robust and translational biomarker.

#### 5.1.2 White matter atrophy

Other morphological modifications in HD patients include atrophy of white matter, cortex, cerebellum and thalamus, as well as global atrophy of the brain (Tabrizi et al., 2009; Della Nave et al., 2010; Paulsen et al., 2010; The TRACK-HD Investigators et al., 2013). Atrophy of the corpus callosum is observed in presymptomatic phase and seems to be the second most affected structure, after striatum.

### 5.2 Metabolic modifications in HD patients

#### 5.2.1 NAA decrease

Metabolic modifications have been extensively studied in HD patients using ^1^H-MRS. Lowered level of tNAA in the striatum is the most common findings, appearing early after or even before the onset of symptoms (Sturrock et al., 2010, 2015; van den Bogaard et al., 2014). Creatine was also reported to be decreased in the caudate of symptomatic patients (den Bogaard et al., 2011; van den Bogaard et al., 2014), as well as in the striatum of juvenile forms of HD (Reynolds et al., 2008). Heterogeneity of HD patients’ metabolic profiles induced contradictory results for other metabolites (Reynolds et al., 2005). Glu and myo-Ins signals in striatum seem to be affected, but both increase and decrease were reported (Sturrock et al., 2010; den Bogaard et al., 2011; van den Bogaard et al., 2014; Sturrock et al., 2015).

NAA decrease and striatal atrophy support the existence of neuronal degeneration, not necessarily leading to neuronal death, that are associated with neuronal shrinkage. It is thus an interesting biomarker for assessing the effect of an experimental treatment in HD models.

#### 5.2.2 Homeostatic processes

Other metabolites showing variable levels depending on mice or human age may indicate compensatory strategies. This theory is also supported by findings of increased ATP production rate in HdhQ111 mice by ^31^P-MRS (Tkac et al., 2012). As discussed, this observation is consistent with works showing local defects of ATP in synapses due to mitochondrial damages (Orr et al., 2008). In clinical HD, ATP and ATP production rate were normal but inorganic Phosphate/ATP ratios as well as inorganic Phosphate /PCr ratios were lowered in response to visual stimulation (Mochel et al., 2012), showing that normal metabolic profile may actually hide abnormal dynamic of energy metabolism. CEST-MRI can provide an alternative to MRS in order to assess metabolic modifications with a better resolution. Several studies have shown the potential of CEST imaging of glutamate to highlight early alteration in small brain structures in HD animal such as corpus callosum. As CEST-MRI can be transferred to clinical scanners, it would be interesting to investigate possible alterations in presymptomatic and HD patients.

### 5.3 Microstructural modifications in HD patients

#### 5.3.1 Diffusion tensor imaging

Microstructural modifications in clinical HD have been thoroughly investigated through DTI. FA value has been shown to decrease in white matter of HD patients (Weaver et al., 2009; Della Nave et al., 2010; Poudel et al., 2015), with corpus callosum being affected even before the onset of symptoms (Rosas et al., 2010). In contrast, FA was measured increased in grey matter, notably in the striatum and globus pallidum (Douaud et al., 2009; van den Bogaard et al., 2012). In the same regions, AD showed increased dispersion, highlighting a sensitivity of the neurons of the striato-pallidal pathways to neuronal damage (Douaud et al., 2009). These results may imply neurodegeneration, neurons shrinkage, or myelin damage (Phillips et al., 2013, 2016; Bourbon-Teles et al., 2019).

#### 5.3.2 Advanced diffusion models

Recently, an effort has been made to use more advanced models to extract more meaningful parameters from diffusion-MRI, such as axonal density. Fixel-based analysis in early-phase HD patients allowed to detect specifically affected fiber bundles with reduced cross-section (FC) but stable fiber density (FD) (Adanyeguh et al., 2021). Though, FD reduction was observed in the fornix (Oh et al., 2021). Interestingly, the same kind of analysis applied to cortico-striatal and cortico-thalamic tracts was able to detect reduction of the fiber density and cross section (FDC) parameter in gene carriers 11 years from onset, while they were still preserved 25 years from onset (Zeun et al., 2022), highlighting the ability of diffusion-MRI to visualize the progression of the disease.

#### 5.3.3 Magnetization transfer imaging

As MT signal is correlated with myelin concentration in tissues, MT imaging can be of good relevance to help identifying microstructural modifications in grey or white matter. While one review reported conflicting results in HD138, several studies observed decreased MT signal in grey matter, including cortex, putamen, hippocampus, and amygdala of patients (Ginestroni et al., 2010; van den Bogaard et al., 2013). More recently, quantitative MT imaging revealed decreased MacroMolecular Proton Fraction (MMPF) in white matter and basal ganglia (Bourbon-Teles et al., 2019). Decreased MT can be interpreted as a reduction in the free water capacity to exchange magnetization with tissues, indicating tissue damage such as demyelination (Tambasco et al., 2015).

Comprehensive interpretation of this corpus seems to point out to a bi-phasic effect of HD mutation on the white matter. Indeed, a model of pathogenesis with initial increase in MT ratio due to compensatory mechanisms (Myers et al., 1991; Johnson et al., 2021), followed by demyelination that seems to play a more important role in the diffusion parameters change than axonal density reduction (Casella et al., 2022). Myelin sheets thickness, as measured using g-ratio, has been reported to be decreased in HD mice models (Xiang et al., 2011; Jin et al., 2015; Teo et al., 2016; Gatto et al., 2019). It was also found in clinical HD with correlation to diffusion parameters (Bourbon-Teles et al., 2019). Additional evidence of white matter impairment was observed with iron content reduction in early-HD patients, using T2*-weighted MRI (di Paola et al., 2014). The use of NODDI and tract reconstruction techniques, as already used in mouse models of HD (Gatto et al., 2021; Gatto and Weissmann, 2022), could help characterizing these white matter abnormalities in prodromal HD.

### 5.4 Functional modifications in HD patients

The measurement of BOLD signal revealed a decrease of activity in cortex but an increase in other regions (Paulsen, 2009). Resting state fMRI unveiled loss of functional connectivity between BOLD-activated regions (Thiruvady et al., 2007; Quarantelli et al., 2013; Wolf et al., 2014), notably in the Default Mode Network (Wolf et al., 2012). Connectivity was also reported to be reduced between motor cortex and caudate of presymptomatic gene carriers (Unschuld et al., 2012). Functional modifications thus seem to appear early in HD. Interestingly, while inter-regions connectivity is decreased, intra-region connectivity was reported to be increased in HD patients (Werner et al., 2014). Dynamic fMRI described a similar mechanism in presymptomatic gene carriers (Aracil-Bolaños et al., 2022). Higher frequency of connections between the Default Mode Network and the central executive network was observed, but reduced variability in these connections, in correlation with cognitive decline. In addition, differences in graph theory-based metrics were also observed, highlighting more random and less efficient connectivity (Gargouri et al., 2016; Aracil-Bolaños et al., 2022).

Dynamic resting state analysis may be able to detect more subtle changes in the mouse brain connectivity due to sensitivity to dynamically changing networks and would be of great interest to implement in future studies. A connection of interest seems to be the cortico-striatal tract, as it seems to be vulnerable in the pathogenesis (Rebec, 2018) and affected early (Unschuld et al., 2012).

### 5.5 Vascular modifications in HD patients

Vascular alterations have been evidenced using MRI. Reduction of the rCBF has been observed in early HD patients (Chen et al., 2012), while regional dependency was discovered in presymptomatic gene carriers (Wolf et al., 2011). Regional CBF seems to be heterogeneously altered, with increase in the precuneus and hippocampus and decrease in the putamen. Increase of arterial CBV was measure with both ASL (Drouin-Ouellet et al., 2015) and iVASO (Hua et al., 2014).

ASL and dynamic contrast enhancement were used to show similar blood brain barrier defects in the R6/2 mouse model and in patients (Drouin-Ouellet et al., 2015). Finally, similar hyperperfusion in patients and R6/2 mice were observed (Lin et al., 2013), in correlation with reduced vascular reactivity. Perfusion MRI is thus a promising tool for the research of biomarkers in HD. This technique could have the potential to report astrocytic impairments as already mentioned in HD88.

## 6 Conclusion

The identification of suitable biomarkers is more than ever needed for studying Huntington’s disease pathogenesis and for evaluation of experimental therapeutic approaches. As illustrated in this review, MRI is able to provide a wide range of biomarkers by using various modalities. In particular, the use of rodent MRI with mouse models of HD is a very efficient way to investigate large aspects of the disease.

This review highlights the importance of choice of the most suitable mouse model and the appropriate MRI/S modality to investigate the pathology. Longitudinal, multiparametric studies are particularly interesting, as the time course of several biomarkers can be very informative and can provide a global characterization of a model, including both morphological, metabolic, microstructural, functional or vascular aspects.

However, the clinical feasibility of a technique should always be kept in mind, as biomarkers found in HD models needs to be transferred to clinical MRI setup to the benefit of patients.

## Author contributions

J-BP: Conceptualization, Writing – original draft. EB: Funding acquisition, Writing – review & editing. JF: Conceptualization, Investigation, Supervision, Writing – review & editing.
